# Combining Theoretical and Experimental Techniques to Study Murine Heart Transplant Rejection

**DOI:** 10.3389/fimmu.2016.00448

**Published:** 2016-11-07

**Authors:** Julia C. Arciero, Andrew Maturo, Anirudh Arun, Byoung Chol Oh, Gerald Brandacher, Giorgio Raimondi

**Affiliations:** ^1^Department of Mathematical Sciences, Indiana University-Purdue University Indianapolis, Indianapolis, IN, USA; ^2^Vascularized and Composite Allotransplantation Laboratory, Department of Plastic and Reconstructive Surgery, Johns Hopkins School of Medicine, Baltimore, MD, USA

**Keywords:** mathematical model, transplant, rejection, immune response, antigen-presenting cells, T cells, cytokines

## Abstract

The quality of life of organ transplant recipients is compromised by complications associated with life-long immunosuppression, such as hypertension, diabetes, opportunistic infections, and cancer. Moreover, the absence of established tolerance to the transplanted tissues causes limited long-term graft survival rates. Thus, there is a great medical need to understand the complex immune system interactions that lead to transplant rejection so that novel and effective strategies of intervention that redirect the system toward transplant acceptance (while preserving overall immune competence) can be identified. This study implements a systems biology approach in which an experimentally based mathematical model is used to predict how alterations in the immune response influence the rejection of mouse heart transplants. Five stages of conventional mouse heart transplantation are modeled using a system of 13 ordinary differential equations that tracks populations of both innate and adaptive immunity as well as proxies for pro- and anti-inflammatory factors within the graft and a representative draining lymph node. The model correctly reproduces known experimental outcomes, such as indefinite survival of the graft in the absence of CD4^+^ T cells and quick rejection in the absence of CD8^+^ T cells. The model predicts that decreasing the translocation rate of effector cells from the lymph node to the graft delays transplant rejection. Increasing the starting number of quiescent regulatory T cells in the model yields a significant but somewhat limited protective effect on graft survival. Surprisingly, the model shows that a delayed appearance of alloreactive T cells has an impact on graft survival that does not correlate linearly with the time delay. This computational model represents one of the first comprehensive approaches toward simulating the many interacting components of the immune system. Despite some limitations, the model provides important suggestions of experimental investigations that could improve the understanding of rejection. Overall, the systems biology approach used here is a first step in predicting treatments and interventions that can induce transplant tolerance while preserving the capacity of the immune system to protect against legitimate pathogens.

## Introduction

Organ transplantation is a life-saving surgical procedure through which the functionality of a failing organ can be restored via replacement with a functioning one. Transplants are performed for a wide variety of organs, including skin, heart, kidney, liver, pancreas, spleen, and lung (1). However, without the administration of immunosuppressive drugs, the recipient’s immune system recognizes the transplanted tissue as a foreign and potentially dangerous material and responds with a massive immune attack that ultimately destroys the graft. This immune response represents a major roadblock in the development of effective therapeutic regimens for the care of patients requiring organ transplants. Current therapeutic regimens rely on chronic immunosuppression. However, the quality of life of transplant recipients is compromised by complications that derive from life-long immunosuppression (such as hypertension, diabetes, opportunistic infections, and cancer) and by the limited long-term graft survival rates due to the absence of established immune tolerance to the transplanted tissues. Ultimately, 20% or more of transplanted patients die by 5 years post-transplant. Thus, there is a pressing need for a new investigative approach to understand the systemic effects that arise from the dynamic interactions between components of the immune system and transplanted tissues.

Previous hypothesis-driven research has provided important insight into the complex interactions among the multiple components of the immune system, including T cells, antigen-presenting cells (APCs), and cytokines (2–10). Such studies have helped to determine the critical players and processes in transplant rejection. For example, the rationale for “costimulation blockade” therapies stems from such studies. These therapies, which target a key step of lymphocyte activation, aim to control T cell activation and promote transplant survival. They have been shown to be a potent strategy for promoting long-term acceptance of transplants in rodents (11–15) and primates (16–18). However, their clinical translation encountered serious difficulties, and ultimately costimulation blockade therapies were only approved as maintenance therapies (19) since they could not promote tolerance (20). To date, the only clinically successful avenue of transplant tolerance induction has been through protocols that induce hematopoietic chimerism (21, 22) (the coexistence of donor and recipient hematopoietic cells) via donor bone marrow co-transplantation with the organ to be transferred. This procedure requires heavy conditioning of patients and carries a significant risk of immunological complications (e.g., the development of graft versus host disease). Consequently, this approach is applicable only in a very restricted cohort of patients in need of a transplant. Thus, a valid and widely applicable strategy to alter the reactivity of the immune system of transplant recipients in a robust and reliable way is still needed.

Biological studies of rejection face various challenges. Experimental *in vivo* models of immune rejection can elucidate precise information regarding select immune cell dynamics and the production and distribution of cytokines. However, conclusions about the system as a whole and the generalizability of these conclusions to other species or types of allografts are further complicated by factors such as procedural variability between models of rejection and variability in parameter measurements. These factors, in combination with the complexity of the immune response, motivate the use of an integrated theoretical and experimental approach to unravel the inter-connected components of the immune response that contribute to transplant rejection. A mathematical model of allograft rejection, refined by multiple clinical and experimental observations, can help to identify variables and parameters that play a significant role in immune system dynamics and yield a better understanding of the complex mechanisms of transplant rejection.

Several computational models have been implemented to predict the dynamics of the immune system in response to viral or bacterial infections (23–26), although only a few theoretical studies have addressed transplant rejection. A recent publication used agent-based modeling (ABM) to investigate solid organ transplant rejection (27). In their study, the model provides an abstract representation of the innate and adaptive immune components involved in the acute rejection process of a solid organ transplant. The study does not use experimentally based parameter values, but it gives a range of possible responses to a transplant challenge without replicating a specific disease process. Another recent study (28) used ordinary differential equations to model the impact of the initial inflammatory response to a surgical insult on overall graft damage. These studies have addressed general transplant immunology questions and have studied a very specific aspect of the initiation of the transplant rejection response, but they do not offer the capacity to capture the important intricacies of the rejection response in a combined experimental and theoretical system that could lead to useful predictions to design new experimentations. The mathematical model presented in the current study aims to provide useful theoretical predictions of transplant rejection based on biologically relevant parameter values, initial conditions, and cellular interactions.

The objectives of this study are (i) to develop a theoretical model to predict the effect of the immune response dynamics on the rejection of a murine heart transplant based on experimental measurements, and (ii) to identify new and effective strategies to promote transplant acceptance that could be investigated experimentally. The model is composed of a system of ordinary differential equations describing the cellular dynamics in the lymph node and graft in the context of a simulated acute rejection of murine heart allograft. The model equations and parameters are based on previous immune system models and are designed to incorporate key assumptions and experimental observations of the immune response to murine heart transplants. The model captures the known behavior of mouse heart rejection and recapitulates the effect of previously reported experimental manipulations. It also underscores the relative importance of the ratios of effector versus regulatory T cells (Tregs) on the speed of graft rejection. Importantly, the model predicts a previously unappreciated behavior when altering the timing of T cell exposure to the graft, providing details for the design of new experimentations that could confirm or refute these findings. Ultimately, we believe this model could become an innovative tool to improve our understanding of transplant rejection and significantly aid in the design of new and effective strategies of immune intervention.

## Materials and Methods

### Model Development

In this study, a mathematical model of murine heart transplants is developed to investigate the interactions between the host immune system and transplanted graft. A compartmental model is used in which all interactions are assumed to occur in either the graft or the draining lymph node. A separate compartment for blood is not included, but the rates of exit and entry of the various cells into the graft or lymph node are assumed to account for transit time in the blood. Table 1 provides the definition and description of all the variables tracked by this model. As with any model, some assumptions and simplifications are necessary to address a specific question using quantitative techniques.

**Table 1:** **Description of model variables**.

Variable	Description	Location
${\text{A}}_{\text{mat}}^{\text{LN}}$	Mature antigen-presenting cells	Lymph node
${\text{T}}_{\text{E}}^{\text{LN}}$	Activated effector T cells	
${\text{T}}_{\text{R}}^{\text{LN}}$	Activated regulatory T cells	
${\text{T}}_{\text{H}}^{\text{LN}}$	Activated helper T cells	
${\text{A}}_{\text{mat}}^{\text{G}}$	Mature antigen-presenting cells	Graft
${\text{A}}_{\text{imm}}^{\text{G}}$	Immature antigen-presenting cells	
${\text{A}}_{\text{inf}}^{\text{G}}$	Inflammatory antigen-presenting cells	
${\text{T}}_{\text{E}}^{\text{G}}$	Activated effector T cells	
${\text{T}}_{\text{R}}^{\text{G}}$	Activated regulatory T cells	
${\text{T}}_{\text{H}}^{\text{G}}$	Activated helper T cells	
G^G^	Graft cells	
${\text{C}}_{\text{p}}^{\text{G}}$	Pro-inflammatory cytokine	
${\text{C}}_{\text{a}}^{\text{G}}$	Anti-inflammatory cytokine	

The following list provides a summary of the assumptions made in this study:

- *Antigen-presenting cells*: a single population of APCs is defined in the model and includes the populations of dendritic cells, macrophages, and B cells; no distinction is made between the origin of the APCs (donor or recipient);
- *Antigen presentation*: direct, indirect, and semi-direct antigen presentation pathways are grouped into a single function;
- *Rejection mechanisms*: only cell-mediated mechanisms of graft cell destruction (by effector T cells and inflammatory APCs) are included since the absence of B cells and associated antibody production in mouse heart transplant models (obtained via genetic manipulation or depletion strategies) does not extend graft survival (29);
- *Lymphoid tissue*: the activation of the immune response is restricted to an ideal lymph node that drains the graft. The contribution of the response by multiple lymphoid tissues is accounted for by amplifying the translocation rate of activated T cells from a single lymph node to the graft;
- *Naïve T cells*: the population of graft-reactive T cells is considered to be homogeneously naïve. No contribution of memory T cells is considered at this stage. A continuous output of newly generated T cells (from the thymus) is assumed to maintain a constant number of naïve T cells in the lymph node. Of the total T cell population, 5% are considered to be alloreactive (30);
- *Inflammatory response*: the danger signals generated by the surgical procedure of transplantation (e.g., surgical trauma, ischemia/reperfusion injury, and potential exposure to bacterial and viral components) (31) and the ensuing release of inflammatory cytokines (i.e., IL-6, IL-18, TNF, IP-10, IL-1) by graft tissues and innate immune components are simplified and represented in a single population $\left({\text{C}}_{\text{p}}^{\text{G}}\right)$. No contribution to the rejection response by concomitant protective immune responses (anti-pathogens) is assumed. Moreover, as no specific quantification of each of inflammatory factors is currently available in the literature, their functions and behavior are represented with a single model variable. ${\text{C}}_{\text{p}}^{\text{G}}$ has both inflammatory and chemotactic functions, and its behavior is initially modeled based on the production and accumulation of IL-6, the most representative inflammatory cytokine in transplantation as previously reported (32, 33);
- *Anti-inflammatory response*: the anti-inflammatory cytokines (i.e., IL-10, TGF-β, IL-35, pro-resolving mediators) normally produced by graft tissues and cells of the immune system as compensatory mechanisms to the inflammatory response initiated by the transplant and by the rejection response are all included in a single population $\left({\text{C}}_{\text{a}}^{\text{G}}\right)$. As per ${\text{C}}_{\text{p}}^{\text{G}}$, there is no transplant-specific quantification available for these factors and ${\text{C}}_{\text{a}}^{\text{G}}$ is mainly modeled on the behavior reported for IL-10 in the regulation of immune responses (34–36).
- *Graft cells turnover*: the growth of heart cells is considered negligible in the model based on reported data (37).

**Figure 1:**
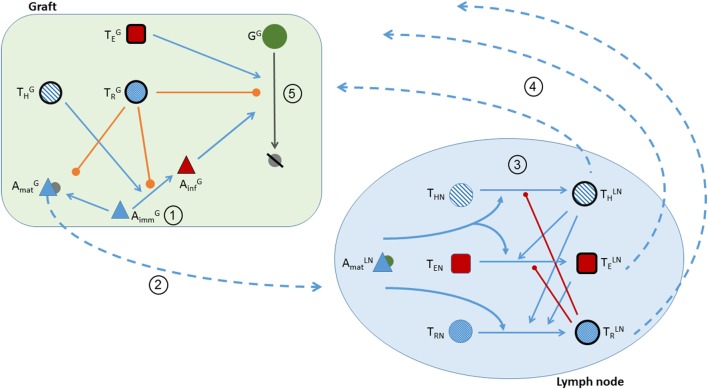
**Schematic of the five stages of transplant rejection defined in the theoretical model**. Circled numbers correspond to the stage number. (1) Transplantation is represented in the model by positive initial conditions for the graft population, pro-inflammatory cytokines, and immature APCs. (2) The activation of ${\text{A}}_{\text{imm}}^{\text{G}}$ into ${\text{A}}_{\text{mat}}^{\text{G}}$ by pro-inflammatory factors, and the translocation of ${\text{A}}_{\text{mat}}^{\text{G}}$ from the graft to the lymphoid tissue. Pro- $\left({\text{C}}_{\text{p}}^{\text{G}}\right)$ and anti-inflammatory $\left({\text{C}}_{\text{a}}^{\text{G}}\right)$ cytokines attract ${\text{A}}_{\text{imm}}^{\text{G}}$ to the graft. (Note the depictions of ${\text{C}}_{\text{a}}^{\text{G}}$- and ${\text{C}}_{\text{p}}^{\text{G}}$-dependent interactions are not included in the figure to prevent overcrowding the schematic. Please refer to the equations for a detailed explanation of the cytokine actions.) (3) The activation of ${\text{T}}_{\text{E}}^{\text{LN}}$, ${\text{T}}_{\text{H}}^{\text{LN}}$, and ${\text{T}}_{\text{R}}^{\text{LN}}$ in the lymph node. T cell activation is facilitated (blue lines) by ${\text{A}}_{\text{mat}}^{\text{LN}}$ and ${\text{T}}_{\text{H}}^{\text{LN}}$ and inhibited (red lines) by ${\text{T}}_{\text{R}}^{\text{LN}}$. (4) The translocation of T cells from the lymph node to the graft. (5) In the presence of ${\text{T}}_{\text{H}}^{\text{G}}$, ${\text{A}}_{\text{imm}}^{\text{G}}$ are activated into ${\text{A}}_{\text{inf}}^{\text{G}}$ (blue line). Graft cells are destroyed by ${\text{A}}_{\text{inf}}^{\text{G}}$ and ${\text{T}}_{\text{E}}^{\text{G}}$. Graft destruction and ${\text{A}}_{\text{imm}}^{\text{G}}$ activation are inhibited by ${\text{T}}_{\text{R}}$ (orange lines).

With these assumptions, the dynamics between the immune system and the graft are described in the following five stages (and are depicted in Figure 1):

1. *Transplantation*: transplantation (introduction of the graft) occurs at day 0 and is captured by the model using the following initial conditions (listed in Table 2) for the graft population (G^G^), pro-inflammatory cytokines $\left({\text{C}}_{\text{p}}^{\text{G}}\right)$, and immature APCs $\left({\text{A}}_{\text{imm}}^{\text{G}}\right)$: G^G^(0) = 5,600,000 cells (38, 39), ${\text{C}}_{\text{p}}^{\text{G}}$ (0) = 50 pg/ml (32, 33), and ${\text{A}}_{\text{imm}}^{\text{G}}$ = 2,000 cells (40, 41). G^G^(0) was chosen by extrapolating the number of cells in a mouse heart based on the average mass of a mouse heart and the average cell density of a human heart. Pro-inflammatory cytokines are assumed to be present at time 0 since transplantation is associated with surgical trauma and exposure to bacterial and viral agents. Additionally, during the procedure, the reconnection to the recipient circulation initiates the process of ischemia/reperfusion injury, causing rapid accumulation of inflammatory mediators (31, 42, 43). Although there is a general agreement on the presence of inflammatory elements at time 0, the overall amount is not known and, thus, an arbitrary value is chosen here. This value reflects the kinetics of mRNA expression and plasma accumulation of IL-6 – a key “danger signal” in the activation of the immune system in transplantation – that have been described previously (32, 33). The presence of these inflammatory cytokines leads to a rapid influx of host immature APCs into the graft (representing the influx of circulating monocytes rapidly converting in the tissues into APCs).
2. *APC maturation and presentation of donor antigens in the lymph node*: once exposed to ${\text{C}}_{\text{p}}^{\text{G}}$, immature APCs are activated into mature APCs in the graft $\left({\text{A}}_{\text{mat}}^{\text{G}}\right)$. The maturation of APCs contributes to an increased accumulation of pro-inflammatory factors as well as, in a delayed fashion, to the production of anti-inflammatory factors $\left({\text{C}}_{\text{a}}^{\text{G}}\right)$ (34–36). Once mature, APCs exit the graft and travel to the draining lymphoid tissue.
3. *Activation of T cells in the lymph node*: in the theoretical model of the lymph node, naïve CD8^+^ effector T cells (T_EN_), naïve Tregs (T_RN_), and naïve CD4^+^ helper T cells (T_HN_) that have the capacity to recognize donor antigens are assumed to be present initially at background levels of 55,000, 9,500, and 70,000 cells, respectively. Upon entering the lymph node, ${\text{A}}_{\text{mat}}^{\text{LN}}$ facilitate the activation of T cells (6, 10). As shown in Figure 1, ${\text{A}}_{\text{mat}}^{\text{LN}}$ are necessary to promote the activation of naïve CD8^+^ $\left({\text{T}}_{\text{E}}^{\text{LN}}\right)$, CD4^+^ $\left({\text{T}}_{\text{H}}^{\text{LN}}\right)$, and regulatory $\left({\text{T}}_{\text{R}}^{\text{LN}}\right)$ T cells in the lymph node. CD8^+^ T cell activation is dependent on the licensing of interacting APCs by activated CD4^+^ T cells. Once activated, Tregs inhibit the activation of CD8^+^ and CD4^+^ T cells. T cell proliferation in the lymph node depends on the autocrine and paracrine effects of growth factors (e.g., IL-2). Tregs are unable to produce and secrete these growth factors and, thus, their proliferation is delayed and dependent on the presence of activated CD4^+^ and CD8^+^ T cells (44).
4. *T cell infiltration of the graft*: following their activation, ${\text{T}}_{\text{E}}^{\text{LN}}$, ${\text{T}}_{\text{H}}^{\text{LN}}$, and ${\text{T}}_{\text{R}}^{\text{LN}}$ exit the lymph node and search for the inflamed tissues of the graft. It is important to note that not all T cells exiting the lymph node will locate the graft. Also, though T cells originate from multiple lymph nodes, only one lymph node is explicitly depicted and described in the model for simplicity. The translocation rate parameters e_E_, e_H_, and e_R_ in Eqs 8–10 are multiplied by a factor k that accounts for the contribution of these two phenomena to the number of cells entering the graft. Specifically, the parameter k is interpreted as the product of the percent of T cells that reach the graft and the number of lymph nodes (and spleen) from which T cells originate.
5. *Destruction of the graft*: in the graft, ${\text{T}}_{\text{H}}^{\text{G}}$ promote the conversion of ${\text{A}}_{\text{imm}}^{\text{G}}$ into inflammatory APCs $\left({\text{A}}_{\text{inf}}^{\text{G}}\right)$; this represents the activation of macrophages into inflammatory cells that release cytotoxic agents (e.g., reactive oxygen species) that induce death of surrounding graft cells. This process is inhibited by ${\text{T}}_{\text{R}}^{\text{G}}$ (45, 46). The release of pro-inflammatory cytokines is promoted in the presence of ${\text{A}}_{\text{mat}}^{\text{G}}$, ${\text{T}}_{\text{E}}^{\text{G}}$, ${\text{T}}_{\text{H}}^{\text{G}}$, and ${\text{A}}_{\text{inf}}^{\text{G}}$. The release of ${\text{C}}_{\text{a}}^{\text{G}}$ is assumed to depend on ${\text{A}}_{\text{mat}}^{\text{G}}$, ${\text{T}}_{\text{R}}^{\text{G}}$, ${\text{C}}_{\text{p}}^{\text{G}}$, G^G^, and ${\text{A}}_{\text{inf}}^{\text{G}}$ (47). The presence of ${\text{C}}_{\text{a}}^{\text{G}}$ inhibits the conversion of ${\text{A}}_{\text{imm}}^{\text{G}}$ into ${\text{A}}_{\text{mat}}^{\text{G}}$ or ${\text{A}}_{\text{inf}}^{\text{G}}$. ${\text{A}}_{\text{inf}}^{\text{G}}$ and ${\text{T}}_{\text{E}}^{\text{G}}$ direct the destruction of the graft, while ${\text{T}}_{\text{R}}^{\text{G}}$ inhibit graft destruction.

**Table 2:** **Initial values for model variables**.

Variable	Initial value	Unit
${\text{A}}_{\text{mat}}^{\text{LN}}$	0	cells
${\text{T}}_{\text{E}}^{\text{LN}}$	0	cells
${\text{T}}_{\text{R}}^{\text{LN}}$	0	cells
${\text{T}}_{\text{H}}^{\text{LN}}$	0	cells
${\text{A}}_{\text{mat}}^{\text{G}}$	200	cells
${\text{A}}_{\text{imm}}^{\text{G}}$	2,000	cells
${\text{A}}_{\text{inf}}^{\text{G}}$	0	cells
${\text{T}}_{\text{E}}^{\text{G}}$	0	cells
${\text{T}}_{\text{R}}^{\text{G}}$	0	cells
${\text{T}}_{\text{H}}^{\text{G}}$	0	cells
G^G^	5.6e6	cells
${\text{C}}_{\text{p}}^{\text{G}}$	50	pg/ml
${\text{C}}_{\text{a}}^{\text{G}}$	0	pg/ml

### Model Equations

The interactions described in the five stages are modeled using a system of 13 ordinary differential equations that tracks cell populations and cytokine concentrations within the graft and lymph node. Many of the parameter values are taken directly from literature sources, some were obtained experimentally, and the remaining are estimated according to experimental assumptions and observations. The initial values of all model variables are given in Table 2. The model parameter values, units, and references are listed in Table 3.

**Table 3:** **Names, values, units, and citations for all model parameters**.

Equation	Parameter name	Value	Unit	Source
1	e_A_	5.5	1/day	(24)
1	μ_A_	1.2	1/day	(48)
2	T_EN_	55,000	cells	Section “Experimental Data Collection”
2	a_E_	3	1/day	(25)
2	γ_1_	100	cells	(25)
2	α_1_	2,500	cells	Estimated
2	μ_E_	0.7	1/day	(25)
2	r_E_	1.51	1/day	(25)
2	β_1_	5,000	cells	Estimated
2	e_E_	0.001	1/day	(24)
3	T_RN_	9,500	cells	Section “Experimental Data Collection”
3	a_R_	2.82e−4	1/day	Optimized
3	γ_2_	1,000	cells	Estimated
3	μ_R_	0.7	1/day	(24, 49)
3	r_R_	0.02	1/day	Estimated
3	α_2_	9,500	cells	Estimated
3	e_R_	0.001	1/day	(24)
4	T_HN_	70,000	cells	Section “Experimental Data Collection”
4	a_H_	6,018.9	cells/day	Optimized
4	γ_3_	100	cells	(25)
4	α_3_	2,500	cells	Estimated
4	μ_H_	0.4	1/day	(24, 25)
4	r_H_	1.51	1/day	(25, 50)
4	γ_4_	4,000	cells	(25)
4	e_H_	0.001	1/day	(24)
5	a_p1_	1,500	cells/day/(pg/ml)	Optimized
5	η_1_	10	pg/ml	Estimated
5	α_4_	12,000	cells	Estimated
6	${\text{k}}_{{\text{C}}_{\text{P}}}$	0.005	1/day/(pg/ml)	Optimized
6	μ_Aimm_	60	1/day	Optimized
6	a_p2_	3.844	1/day	Optimized
6	η_2_	10	pg/ml	Estimated
6	α_5_	12,000	cells	Estimated
7	μ_Ainf_	1.2	1/day	(48)
8	k	15	–	Estimated
8	r_EG_	0.3	1/day	Optimized
8	η_3_	10	pg/ml	Estimated
8	β_2_	4e6	cells	Estimated
9	r_RG_	0.00375	1/day	Optimized
9	α_6_	12,000	cells	Estimated
10	r_HG_	0.755	1/day	Estimated
10	γ_5_	4,000	cells	Estimated
10	η_4_	10	pg/ml	Estimated
11	d_inf_	0.055	1/day	Optimized
11	α_7_	12,000	cells	Estimated
11	d_E_	0.004	cells/day	Optimized
11	α_8_	12,000	cells	Estimated
12	ρ_1_	10.98	(pg/ml)/day	Optimized
12	α_9_	12,000	cells	Estimated
12	ρ_2_	0.024	(pg/ml)/day	Estimated
12	α_10_	12,000	cells	Estimated
12	ρ_3_	0.24	(pg/ml)/day	Estimated
12	α_11_	12,000	cells	Estimated
12	ρ_4_	10.95	(pg/ml)/day	Optimized
12	α_12_	12,000	cells	Estimated
12	$\mu_{{\text{C}}_{\text{p}}}$	0.15	1/day	Optimized
13	ξ_1_	2.08e−4	(pg/ml)/cells/day	Estimated
13	ξ_2_	6.3e−6	(pg/ml)/cells/day	Estimated
13	ξ_3_	4.2e−9	1/cells/day	Estimated
13	ξ_4_	2.5e−4	(pg/ml)/cells/day	Estimated
13	$\mu_{{\text{C}}_{\text{a}}}$	0.05	1/day	Estimated

The model equations describe the activation, proliferation, natural decay, destruction, and inhibition of the various populations when appropriate. The interactions in the lymph node are modeled using four equations, and the interactions in the graft are modeled using nine equations. The superscripts LN and G denote cell populations in the lymph node and graft, respectively.

In Eq. 1, the rate of change of mature APCs in the lymph node $\left({\text{A}}_{\text{mat}}^{\text{LN}}\right)$ is defined. This rate depends on the entrance of mature APCs from the graft at rate e_A_ (first term) and on the natural decay of ${\text{A}}_{\text{mat}}^{\text{LN}}$ in the lymph node (second term).

(1) $$\frac{{\text{dA}}_{\text{mat}}^{\text{LN}}}{\text{dt}}={\text{e}}_{\text{A}}{\text{A}}_{\text{mat}}^{\text{G}}-\mu_{\text{A}}{\text{A}}_{\text{mat}}^{\text{LN}}$$

The naïve alloreactive T cell populations (T_EN_, T_RN_, and T_HN_) in the lymph node are assumed to be constant since a background population of these cells is always present (due to thymopoiesis).

In Eq. 2, the rate of change in CD8^+^ T cells in the lymph node is shown to depend on T cell activation (term 1), decay (term 2), proliferation (term 3), and translocation (term 4). The activation of ${\text{T}}_{\text{E}}^{\text{LN}}$ depends on the presence of both ${\text{A}}_{\text{mat}}^{\text{LN}}$ and ${\text{T}}_{\text{H}}^{\text{LN}}$, while ${\text{T}}_{\text{R}}^{\text{LN}}$ inhibit this process (10, 46, 51). Proliferation of the ${\text{T}}_{\text{E}}^{\text{LN}}$ cells depends on ${\text{A}}_{\text{mat}}^{\text{LN}}$ and ${\text{T}}_{\text{E}}^{\text{LN}}$ and occurs at rate r_E_ (used as a proxy for the production, secretion, and autocrine effect of IL-2) (52). The translocation of ${\text{T}}_{\text{E}}^{\text{LN}}$ from the lymph node is assumed to occur at rate e_E_.

(2) $$\frac{{\text{dT}}_{\text{E}}^{\text{LN}}}{\text{dt}}=\frac{{\text{a}}_{\text{E}}{\text{T}}_{\text{EN}}{\text{A}}_{\text{mat}}^{\text{LN}}{\text{T}}_{\text{H}}^{\text{LN}}}{\left(\gamma_{1}+{\text{A}}_{\text{mat}}^{\text{LN}}\right)\left(\alpha_{1}+{\text{T}}_{\text{R}}^{\text{LN}}\right)}-\mu_{\text{E}}{\text{T}}_{\text{E}}^{\text{LN}}+\frac{{\text{r}}_{\text{E}}{\text{T}}_{\text{E}}^{\text{LN}}{\text{A}}_{\text{mat}}^{\text{LN}}}{\beta_{1}+{\text{A}}_{\text{mat}}^{\text{LN}}}-{\text{e}}_{\text{E}}{\text{T}}_{\text{E}}^{\text{LN}}$$

The rates of change of Tregs in the lymph node (Eq. 3) and CD4^+^ T cells in the lymph node (Eq. 4) contain the same four terms (activation, decay, proliferation, and exit) as the equation for CD8^+^ T cells, with a few important differences: the activation of ${\text{T}}_{\text{R}}^{\text{LN}}$ and ${\text{T}}_{\text{H}}^{\text{LN}}$ depends only on ${\text{A}}_{\text{mat}}^{\text{LN}}$, and the proliferation of ${\text{T}}_{\text{R}}^{\text{LN}}$ occurs only in the presence of ${\text{T}}_{\text{E}}^{\text{LN}}$ or ${\text{T}}_{\text{H}}^{\text{LN}}$. The activation of ${\text{T}}_{\text{H}}^{\text{LN}}$ is inhibited by ${\text{T}}_{\text{R}}^{\text{LN}}$ (10, 45).

(3) $$\frac{{\text{dT}}_{\text{R}}^{\text{LN}}}{\text{dt}}=\frac{{\text{a}}_{\text{R}}{\text{T}}_{\text{RN}}{\text{A}}_{\text{mat}}^{\text{LN}}}{\gamma_{2}+{\text{A}}_{\text{mat}}^{\text{LN}}}-\mu_{\text{R}}{\text{T}}_{\text{R}}^{\text{LN}}+\frac{{\text{r}}_{\text{R}}{\text{T}}_{\text{R}}^{\text{LN}}\left({\text{T}}_{\text{E}}^{\text{LN}}+{\text{T}}_{\text{H}}^{\text{LN}}\right)}{\alpha_{2}+{\text{T}}_{\text{R}}^{\text{LN}}}-{\text{e}}_{\text{R}}{\text{T}}_{\text{R}}^{\text{LN}}$$

(4) $$\frac{{\text{dT}}_{\text{H}}^{\text{LN}}}{\text{dt}}=\frac{{\text{a}}_{\text{H}}{\text{T}}_{\text{HN}}{\text{A}}_{\text{mat}}^{\text{LN}}}{\left(\gamma_{3}+{\text{A}}_{\text{mat}}^{\text{LN}}\right)\left(\alpha_{3}+{\text{T}}_{\text{R}}^{\text{LN}}\right)}-\mu_{\text{H}}{\text{T}}_{\text{H}}^{\text{LN}}+\frac{{\text{r}}_{\text{H}}{\text{T}}_{\text{H}}^{\text{LN}}{\text{A}}_{\text{mat}}^{\text{LN}}}{\gamma_{4}+{\text{A}}_{\text{mat}}^{\text{LN}}}-{\text{e}}_{\text{H}}{\text{T}}_{\text{H}}^{\text{LN}}$$

The rate of change of the ${\text{A}}_{\text{imm}}^{\text{G}}$ population in the graft is defined in Eq. 5. The first term represents the influx of ${\text{A}}_{\text{imm}}^{\text{G}}$ due to the presence of the graft and pro-inflammatory cytokines. The second term accounts for the natural decay of ${\text{A}}_{\text{imm}}^{\text{G}}$. The third term indicates the loss of immature APCs once they become activated into ${\text{A}}_{\text{mat}}^{\text{G}}$. The fourth term defines the ${\text{T}}_{\text{H}}^{\text{G}}$-mediated activation of ${\text{A}}_{\text{imm}}^{\text{G}}$ into ${\text{A}}_{\text{inf}}^{\text{G}}$. In both of these last two terms, the conversion of ${\text{A}}_{\text{imm}}^{\text{G}}$ into activated populations is inhibited by ${\text{C}}_{\text{a}}^{\text{G}}$ and ${\text{T}}_{\text{R}}^{\text{G}}$ (35, 47, 51, 53, 54). The functional form describing the inhibition by ${\text{C}}_{\text{a}}^{\text{G}}$ is chosen to emphasize that the rate is a decreasing sigmoidal function of ${\text{C}}_{\text{a}}^{\text{G}}$.

(5) $$\frac{{\text{dA}}_{\text{imm}}^{\text{G}}}{\text{dt}}={\text{k}}_{{\text{C}}_{\text{p}}}{\text{C}}_{\text{p}}^{\text{G}}{\text{G}}^{\text{G}}-\mu_{\text{Aimm}}{\text{A}}_{\text{imm}}^{\text{G}}-{\text{a}}_{\text{p1}}\left(1-\frac{{\left({\text{C}}_{\text{a}}^{\text{G}}\right)}^{\text{2}}}{\eta_{1}^{2}+{\left({\text{C}}_{\text{a}}^{\text{G}}\right)}^{\text{2}}}\right)\times \left(\frac{{\text{A}}_{\text{imm}}^{\text{G}}{\text{C}}_{\text{p}}^{\text{G}}}{\alpha_{4}+{\text{T}}_{\text{R}}^{\text{G}}}\right)-{\text{a}}_{\text{p2}}\left(1-\frac{{\left({\text{C}}_{\text{a}}^{\text{G}}\right)}^{\text{2}}}{\eta_{2}^{2}+{\left({\text{C}}_{\text{a}}^{\text{G}}\right)}^{\text{2}}}\right)\left(\frac{{\text{A}}_{\text{imm}}^{\text{G}}{\text{T}}_{\text{H}}^{\text{G}}}{\alpha_{5}+{\text{T}}_{\text{R}}^{\text{G}}}\right)$$

Equation 6 describes the dynamics of mature APCs in the graft $\left({\text{A}}_{\text{mat}}^{\text{G}}\right)$. The first term defines the activation of ${\text{A}}_{\text{mat}}^{\text{G}}$ by ${\text{C}}_{\text{p}}^{\text{G}}$, which is inhibited by ${\text{C}}_{\text{a}}^{\text{G}}$ and ${\text{T}}_{\text{R}}^{\text{G}}$ (35, 47, 51, 53, 54). The second term is the natural decay of ${\text{A}}_{\text{mat}}^{\text{G}}$, and the last term accounts for the exit of ${\text{A}}_{\text{mat}}^{\text{G}}$ from the graft to the lymph node.

(6) $$\frac{{\text{dA}}_{\text{mat}}^{\text{G}}}{\text{dt}}={\text{a}}_{\text{p1}}\left(1-\frac{{\left({\text{C}}_{\text{a}}^{\text{G}}\right)}^{\text{2}}}{\eta_{1}^{2}+{\left({\text{C}}_{\text{a}}^{\text{G}}\right)}^{\text{2}}}\right)\left(\frac{{\text{A}}_{\text{imm}}^{\text{G}}{\text{C}}_{\text{p}}^{\text{G}}}{\alpha_{4}+{\text{T}}_{\text{R}}^{\text{G}}}\right)-\mu_{\text{A}}{\text{A}}_{\text{mat}}^{\text{G}}-{\text{e}}_{\text{A}}{\text{A}}_{\text{mat}}^{\text{G}}$$

In Eq. 7, inflammatory APCs are differentiated from ${\text{A}}_{\text{imm}}^{\text{G}}$ in the presence of ${\text{T}}_{\text{H}}^{\text{G}}$ and inhibited by ${\text{C}}_{\text{a}}^{\text{G}}$ and ${\text{T}}_{\text{R}}^{\text{G}}$ (term 1) (35, 47, 51, 53, 54) and are assumed to exhibit natural decay (term 2).

(7) $$\frac{{\text{dA}}_{\text{inf}}^{\text{G}}}{\text{dt}}={\text{a}}_{\text{p2}}\left(1-\frac{{\left({\text{C}}_{\text{a}}^{\text{G}}\right)}^{\text{2}}}{\eta_{2}^{2}+{\left({\text{C}}_{\text{a}}^{\text{G}}\right)}^{\text{2}}}\right)\left(\frac{{\text{A}}_{\text{imm}}^{\text{G}}{\text{T}}_{\text{H}}^{\text{G}}}{\alpha_{5}+{\text{T}}_{\text{R}}^{\text{G}}}\right)-\mu_{\text{Ainf}}{\text{A}}_{\text{inf}}^{\text{G}}$$

The rates of change for CD8^+^, regulatory, and CD4^+^ T cells in the graft (Eqs 8, 9, and 10, respectively) depend on the rate at which they enter the graft (term 1), their natural decay (term 2), and their proliferation (term 3). The proliferation of ${\text{T}}_{\text{E}}^{\text{G}}$ and ${\text{T}}_{\text{H}}^{\text{G}}$ is inhibited by ${\text{C}}_{\text{a}}^{\text{G}}$ (36). The parameter k that multiplies the exit rate of T cells from the lymph node accounts for the fact that not all T cells exiting the lymph node reach the graft and that T cells arrive from multiple lymph nodes. The value for k is obtained from the product of the percent of T cells that reach the graft and the number of lymph nodes from which T cells originate.

(8) $$\frac{{\text{dT}}_{\text{E}}^{\text{G}}}{\text{dt}}=\text{k}{\text{e}}_{\text{E}}{\text{T}}_{\text{E}}^{\text{LN}}-\mu_{\text{E}}{\text{T}}_{\text{E}}^{\text{G}}+{\text{r}}_{\text{EG}}\left(1-\frac{{\left({\text{C}}_{\text{a}}^{\text{G}}\right)}^{\text{2}}}{\eta_{3}^{2}+{\left({\text{C}}_{\text{a}}^{\text{G}}\right)}^{\text{2}}}\right)\frac{{\text{T}}_{\text{E}}^{\text{G}}{\text{G}}^{\text{G}}}{\beta_{2}+{\text{G}}^{\text{G}}}$$

(9) $$\frac{{\text{dT}}_{\text{R}}^{\text{G}}}{\text{dt}}=\text{k}{\text{e}}_{\text{R}}{\text{T}}_{\text{R}}^{\text{LN}}-\mu_{\text{R}}{\text{T}}_{\text{R}}^{\text{G}}+\frac{{\text{r}}_{\text{RG}}{\text{T}}_{\text{R}}^{\text{G}}\left({\text{T}}_{\text{E}}^{\text{G}}{\text{+T}}_{\text{H}}^{\text{G}}\right)}{\alpha_{6}+{\text{T}}_{\text{R}}^{\text{G}}}$$

(10) $$\frac{{\text{dT}}_{\text{H}}^{\text{G}}}{\text{dt}}=\text{k}{\text{e}}_{\text{H}}{\text{T}}_{\text{H}}^{\text{LN}}-\mu_{\text{H}}{\text{T}}_{\text{H}}^{\text{G}}+{\text{r}}_{\text{HG}}\left(1-\frac{{\left({\text{C}}_{\text{a}}^{\text{G}}\right)}^{\text{2}}}{\eta_{4}^{2}+{\left({\text{C}}_{\text{a}}^{\text{G}}\right)}^{\text{2}}}\right)\frac{{\text{T}}_{\text{H}}^{\text{G}}{\text{A}}_{\text{mat}}^{\text{G}}}{\gamma_{5}+{\text{A}}_{\text{mat}}^{\text{G}}}$$

Equation 11 describes the dynamics of the mass of the graft. The first and second terms represent the destruction of the graft due to ${\text{A}}_{\text{inf}}^{\text{G}}$ and ${\text{T}}_{\text{E}}^{\text{G}}$, respectively. ${\text{T}}_{\text{R}}^{\text{G}}$ work to inhibit the destruction of the graft through mechanisms that differ from the ones used in the lymph node. It is recognized that in non-lymphoid tissues, ${\text{T}}_{\text{R}}^{\text{G}}$ do not inhibit the accumulation, nor the proliferation, of ${\text{A}}_{\text{imm}}^{\text{G}}$, ${\text{T}}_{\text{E}}^{\text{G}}$, and ${\text{T}}_{\text{H}}^{\text{G}}$. Instead, they prevent damage via inhibition of the destructive activities of ${\text{A}}_{\text{inf}}^{\text{G}}$ and ${\text{T}}_{\text{E}}^{\text{G}}$ (in addition to preventing the conversion of ${\text{A}}_{\text{imm}}^{\text{G}}$ into ${\text{A}}_{\text{inf}}^{\text{G}}$ and ${\text{A}}_{\text{mat}}^{\text{G}}$, depicted in Eqs. 6 and 7) (54–56). No growth of graft cells is assumed in this model as stated in our model assumptions.

(11) $$\frac{\text{d}{\text{G}}^{\text{G}}}{\text{dt}}=-\frac{{\text{d}}_{\text{inf}}{\text{A}}_{\text{inf}}^{\text{G}}{\text{G}}^{\text{G}}}{\left(\alpha_{7}+{\text{T}}_{\text{R}}^{\text{G}}\right)}-\frac{{\text{d}}_{\text{E}}{\text{T}}_{\text{E}}^{\text{G}}{\text{G}}^{\text{G}}}{\left(\alpha_{8}+{\text{T}}_{\text{R}}^{\text{G}}\right)}$$

As defined in Eq. 12, the release of pro-inflammatory cytokines is triggered by the conversion of ${\text{A}}_{\text{imm}}^{\text{G}}$ into ${\text{A}}_{\text{mat}}^{\text{G}}$ and ${\text{A}}_{\text{inf}}^{\text{G}}$ (terms 1 and 4) as well as by the execution of effector functions by both ${\text{T}}_{\text{E}}^{\text{G}}$ and ${\text{T}}_{\text{H}}^{\text{G}}$ recognizing their target (10) (terms 2 and 3). The release of ${\text{C}}_{\text{p}}^{\text{G}}$ by each of these cells is inhibited by ${\text{T}}_{\text{R}}^{\text{G}}$ (46, 54). The natural decay of ${\text{C}}_{\text{p}}^{\text{G}}$ is modeled in the last term.

(12) $$\frac{{\text{dC}}_{\text{p}}^{\text{G}}}{\text{dt}}=\frac{\rho_{1}{\text{A}}_{\text{mat}}^{\text{G}}}{\alpha_{9}+{\text{T}}_{\text{R}}^{\text{G}}}+\frac{\rho_{2}{\text{T}}_{\text{E}}^{\text{G}}}{\alpha_{10}+{\text{T}}_{\text{R}}^{\text{G}}}+\frac{\rho_{3}{\text{T}}_{\text{H}}^{\text{G}}}{\alpha_{11}+{\text{T}}_{\text{R}}^{\text{G}}}+\frac{\rho_{4}{\text{A}}_{\text{inf}}^{\text{G}}}{\alpha_{12}+{\text{T}}_{\text{R}}^{\text{G}}}-\mu_{{\text{C}}_{\text{p}}}{\text{C}}_{\text{p}}^{\text{G}}$$

Equation 13 describes the release of ${\text{C}}_{\text{a}}^{\text{G}}$ due to the conversion of ${\text{A}}_{\text{imm}}^{\text{G}}$ into ${\text{A}}_{\text{mat}}^{\text{G}}$ and ${\text{A}}_{\text{inf}}^{\text{G}}$ – a regulatory pathway embedded in the process of activation to prevent uncontrolled reactivity (35) – and due to activity of ${\text{T}}_{\text{R}}^{\text{G}}$ (46) that infiltrate the graft (terms 1, 4, and 2, respectively). Upon encountering pro-inflammatory cytokines, the graft tissue also produces anti-inflammatory mediators (term 3). The last term gives the natural decay of ${\text{C}}_{\text{a}}^{\text{G}}$. Since the four populations leading to the production of ${\text{C}}_{\text{a}}^{\text{G}}$ are already inhibited in the presence of ${\text{C}}_{\text{a}}^{\text{G}}$, additional inhibition is not included in any of the terms.

(13) $$\frac{{\text{dC}}_{\text{a}}^{\text{G}}}{\text{dt}}=\xi_{1}{\text{A}}_{\text{mat}}^{\text{G}}+\xi_{2}{\text{T}}_{\text{R}}^{\text{G}}+\xi_{3}{\text{C}}_{\text{p}}^{\text{G}}{\text{G}}^{\text{G}}+\xi_{4}{\text{A}}_{\text{inf}}^{\text{G}}-\mu_{{\text{C}}_{\text{a}}}{\text{C}}_{\text{a}}^{\text{G}}$$

### Experimental Data Collection

Male 8- to 10-week-old Balb/C (H-2^d^), and C57BL/6 (B6; H-2^b^) mice were purchased from the Jackson Laboratory (Bar Harbor, ME, USA) and housed in specific pathogen-free facilities at Johns Hopkins University, Baltimore, MD, USA. All experiments were conducted according to Institutional Animal Care and Use Committee-approved protocols.

Heterotopic (intra-abdominal) heart transplantation was performed from BALB/c to B6 mice, as previously described (57). On day 7 post-transplantation, cells from grafts were isolated using an adaptation of the technique described by Setoguchi et al. (58). Briefly, tissues were digested at 37°C via 3 consecutive 15-min incubations in PBS containing Collagenase IV (560 U/ml; Worthington) DNAse I (275 U/ml; Amresco), and Dispase II (0.4 U/ml; Roche). Leukocytes were enriched using a 24% Histodenz (Sigma-Aldrich)-based gradient separation. These preparations were then used to quantify the content of CD4^+^, CD8^+^, and Tregs in the rejecting hearts via flow cytometry. Cells were stained using anti-CD4^+^ and anti-CD8^+^ mAb (from BD Bioscience) and anti-Foxp3 mAb (Affymetrix/eBioscience) according to the manufacturer protocols; samples were acquired using a BD LSR-II flow cytometer. Data were analyzed via FlowJo analysis software (FlowJo, LLC).

Table 4 summarizes the absolute counts and relative ratios of T cell subsets infiltrating a rejecting heart on post-operative day (POD) 7 deriving from such analysis. From these data, the biological variability observed between animals in the total number of each subset that infiltrate the heart is clearly evident. Strikingly, however, the ratios among T cell subsets were maintained within very narrow ranges. Consequently, we used the average number of T cells to set the scale for the number of T cells in the model, and we optimized various model parameters to the observed ratios of T cells.

**Table 4:** **Absolute counts and relative ratios of T cell subsets infiltrating a rejecting murine heart on POD 7**.

	CD8	CD4	CD8/CD4 ratio	Treg	Treg (% of CD4)
Heart #1	2.7e6	5.4e5	5	7.4e4	13.6
Heart #2	4.3e5	8.3e4	5.3	1.2e4	14.9
Heart #3	6.5e5	1.8e5	3.7	2.5e4	14.1
**Average**	**1.27e6**	**2.7e5**	**4.7**	**3.7e4**	**14.2**
SE	6e5	1.2e5	0.4	1.5e4	0.3

A similar approach was used to determine the average number of each T cell subset in a typical lymph node. Our data agree with a previously published data set (3). Briefly, collection of 16 lymph nodes from multiple animals averaged the identification of 17e6 CD8^+^ T cells, 22e6 CD4^+^ T cells, and 3e6 Treg. This renders 1.1e6 CD8^+^ T cells, 1.4e6 CD4^+^ T cells, and 0.19e6 Treg in the average lymph node. Considering that ~5% of T cells are reactive against donor antigens, the average lymph nodes contains (at time 0) 55,000 CD8^+^ T cells, 70,000 CD4^+^ T cells, and 9,500 Treg.

### Parameter Estimation

The model contains 61 parameters. Many of the values of these parameters have been obtained directly from experimental studies (1–6, 8–10, 26, 32, 33, 46, 47, 50–52, 57, 59–68) or other mathematical models of the immune system (3, 23–26, 48, 69–74). Table 3 provides a list of all the model parameter values and sources for their values when possible. A definition of “estimated” in Table 3 indicates that the value was not found directly in the literature but was estimated according to known relationships and ratios among cell populations in the model. For example, due to the potency and cellular similarities of Tregs and helper T cells, the activation rate of ${\text{T}}_{\text{R}}^{\text{LN}}$ is assumed to be smaller than the activation rate of ${\text{T}}_{\text{H}}^{\text{LN}}$ (46). As another example, the death rate of ${\text{A}}_{\text{inf}}^{\text{G}}$ is assumed to equal the death rate of ${\text{A}}_{\text{mat}}^{\text{G}}$. The constant values for T_EN_ and T_HN_ are obtained from experiments conducted in the present study (Table 4). According to reported ratios (26, 46, 57), the T_RN_ population should be chosen to be about one-tenth of the helper T cell initial populations.

Several remaining model parameters are optimized (and are defined as “optimized” in Table 3) to satisfy the following experimental observations:

(1) Presence of all T cells
  - *APC conditions* (40, 41)
    - ${\text{A}}_{\text{mat}}^{\text{G}}$ have a peak population of ~18,000 cells.
    - ${\text{A}}_{\text{imm}}^{\text{G}}$ have a peak population of ~12,000 cells.
    - ${\text{A}}_{\text{mat}}^{\text{G}}$ and ${\text{A}}_{\text{imm}}^{\text{G}}$ peak between days 1 and 3.
  - *Graft destruction* (66)
    - A 75% reduction of the graft mass occurs by 12–14 days following transplantation.
  - *T cell ratios* (Table 4 and see Experimental Data Collection)
    - The maximum ${\text{T}}_{\text{E}}^{\text{G}}$ value is approximately five times greater than the maximum ${\text{T}}_{\text{H}}^{\text{G}}$ value (ratio of average ${\text{T}}_{\text{E}}^{\text{G}}$:${\text{T}}_{\text{H}}^{\text{G}}$ values is 4.7, Table 4).
    - The maximum ${\text{T}}_{\text{H}}^{\text{G}}$ value is approximately seven times greater than the maximum ${\text{T}}_{\text{R}}^{\text{G}}$ value (ratio of average ${\text{T}}_{\text{H}}^{\text{G}}$:${\text{T}}_{\text{R}}^{\text{G}}$ values is 7.29, Table 4).
    - The maximum number of ${\text{T}}_{\text{E}}^{\text{LN}}$ occurs at ~4 days post-transplantation.
    - The maximum number of ${\text{T}}_{\text{E}}^{\text{G}}$ occurs at ~6 days post-transplantation.
(2) *Absence of helper T cells* (7, 64)
  No graft rejection.
(3) *Absence of effector T cells* (7, 64)
  Rejection should be delayed slightly.
(4) *Absence of all T cells* (40, 41)
  - No damage to the graft.
  - APC measures:
    - Immature APCs: 23000, 12000, 2100, and 2000 cells on days 1, 3, 5, and 10.
    - Mature APCs: 17000, 12000, 2400, 3000 on days 1, 3, 5, and 10.

## Results

### Model Verification

The following four model simulations were used to confirm that the model results indeed reflect the assumptions on which the model was built in terms of expected physiological behavior.

#### Timing of Graft Rejection

**Figure 2:**
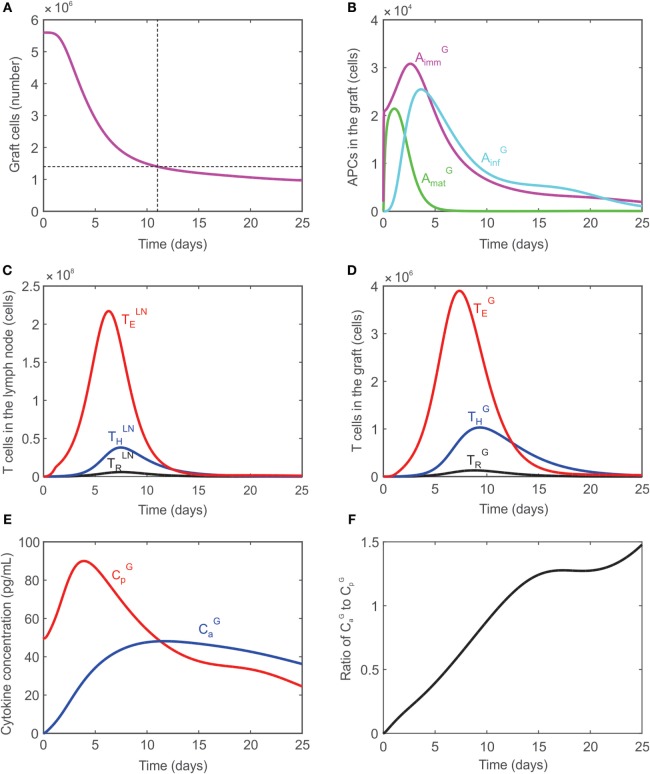
**(A)** Graft rejection is predicted to occur ~11 days following transplantation. **(B)** Model predicted values of immature APCs (${\text{A}}_{\text{imm}}^{\text{G}}$, magenta), mature APCs (${\text{A}}_{\text{mat}}^{\text{G}}$, green), and inflammatory APCs (${\text{A}}_{\text{inf}}^{\text{G}}$, cyan) in the graft. **(C)** Model predicted values of regulatory T cells (black), CD4^+^ T cells (blue), CD8^+^ T cells (red) in the lymph node. **(D)** Model predicted values of regulatory T cells (black), CD4^+^ T cells (blue), CD8^+^ T cells (red) in the graft. **(E)** Model predicted concentration of pro-inflammatory cytokines (${\text{C}}_{\text{p}}^{\text{G}}$, red) and anti-inflammatory cytokines (${\text{C}}_{\text{a}}^{\text{G}}$, blue) in the graft. **(F)** Ratio of ${\text{C}}_{\text{a}}^{\text{G}}$:${\text{C}}_{\text{p}}^{\text{G}}$ in the graft.

In the absence of any external manipulation (i.e., administration of immunosuppressive drugs or any immunomodulatory intervention), experimental murine cardiac transplants are rejected at ~12–14 days after transplantation. Tanaka et al. (66) performed *in vivo* visualization of murine cardiac allograft rejection and identified the cessation of the heartbeat to occur on day 12, which corresponded to a 75% reduction in the measured luminescence of donor tissue from transgenic luciferase-GFP (green fluorescent protein)-modified mice. The present model uses this as an approximate metric, defining graft rejection once the number of graft cells has decreased by 75% of their initial number. Figure 2A shows the time dynamics of graft rejection predicted by the model. The behavior of other key populations including APCs in the graft, T cells in the lymph node, T cells in the graft, and cytokines in the graft are shown in Figures 2B–F. The number of T cells in the lymph node peaks around days 6–7 in the lymph node and days 7–9 in the graft, which agrees with experimental observations (70). The ratios of ${\text{T}}_{\text{E}}^{\text{G}}$:${\text{T}}_{\text{H}}^{\text{G}}$ and ${\text{T}}_{\text{H}}^{\text{G}}$:${\text{T}}_{\text{R}}^{\text{G}}$ at their peaks are calculated to be 4.7 and 7.29, respectively, in the graft, which are consistent with experimental values obtained in this study (Table 4).

#### Graft Rejection in the Absence of CD8^+^ T Cells (T_E_)

**Figure 3:**
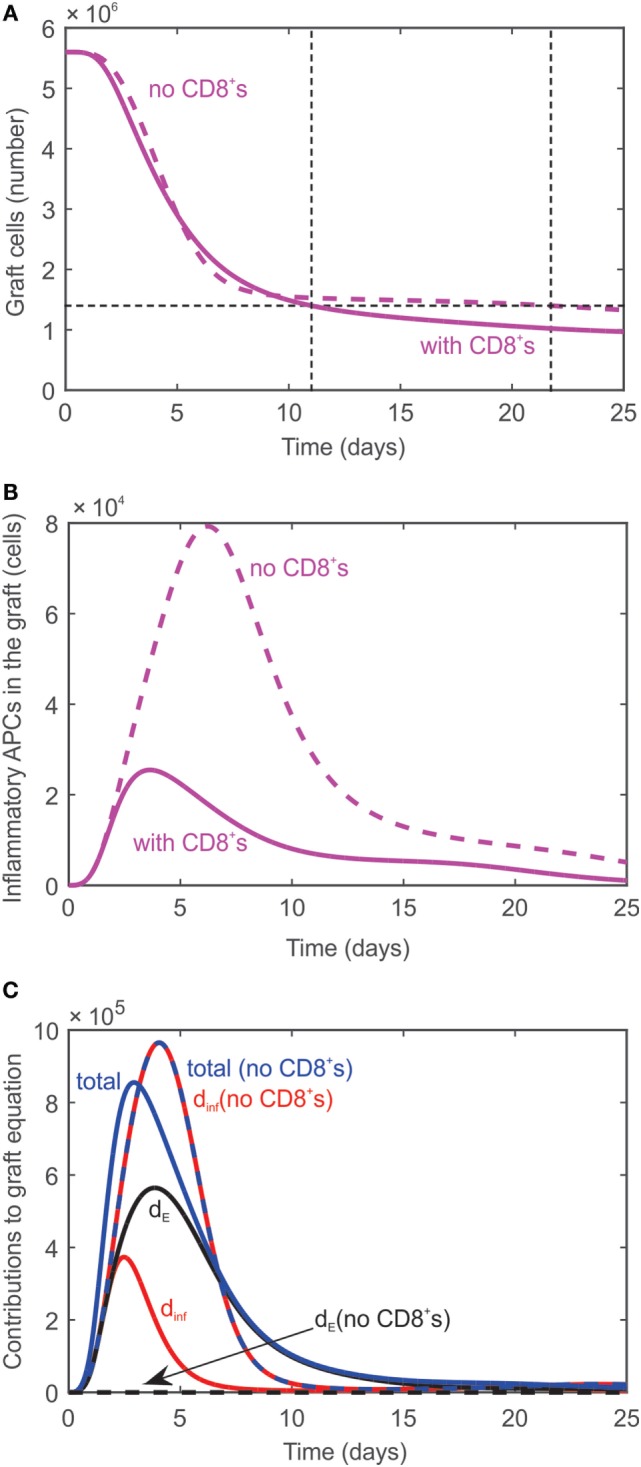
**(A)** Graft rejection is delayed by ~10 days in the absence of CD8^+^ T cells. Dashed curve: model prediction in the absence of effector T cells. Solid curve: model prediction in the presence of all T cells. **(B)** Model predicted values of inflammatory APCs in the absence of effector T cells (dashed curve) and in the presence of all T cells (solid curve). **(C)** Individual contributions of ${\text{A}}_{\text{inf}}^{\text{G}}$ (red curve, labeled d_inf_) and CD8^+^ T cells (black curve, labeled d_E_) and combined contribution (blue curve, labeled total) to the rate of change of the graft population in the absence (dashed) and presence (solid) of CD8^+^ T cells.

As demonstrated experimentally (7), transplant rejection can occur even if no CD8^+^ cells are present in the system; the time to rejection is just slightly delayed. The absence of CD8^+^ cells is simulated in the model by modifying the initial value of naïve effector T cells to be T_EN_ = 0. As a result, no effector T cells are generated in the lymph node, but graft rejection is predicted to occur at day 22 (dashed curve, Figure 3A) instead of day 11 when all T cells are present (solid curve, Figure 3A). Rejection is predicted to occur despite the absence of CD8^+^ T cells since activated CD4^+^ T cells in the graft promote the differentiation of inflammatory APCs (Figure 3B) which cause graft destruction. Figure 3C serves to explain why the graft is not destroyed sooner when no CD8^+^ T cells are present given that ${\text{A}}_{\text{inf}}^{\text{G}}$ is much higher in their absence (Figure 3B). When all T cells are present, the graft is destroyed by both ${\text{A}}_{\text{inf}}^{\text{G}}$ and CD8^+^ T cells (terms 1 and 2 in Eq. 11, respectively). The contribution of each of these terms to the rate of change of the graft population is plotted in Figure 3C (solid curves). Specifically, the contribution of the ${\text{A}}_{\text{inf}}^{\text{G}}$ term (labeled d_inf_) is shown in red, the contribution of the CD8^+^ T cells when they are present (labeled d_E_) is shown in black, and the sum of these contributions (labeled total) is shown in blue. The dashed curves correspond to these same cases when CD8^+^ T cells are absent. Note that in this case the contribution of ${\text{A}}_{\text{inf}}^{\text{G}}$ (dashed red curve) and the sum of the contributions (dashed blue curve) lie on top of each other since the contribution of the CD8^+^ T cells is zero (black dashed curve). As can be seen in Figure 3C, the contribution of ${\text{A}}_{\text{inf}}^{\text{G}}$ when CD8^+^ T cells are absent exceeds the total solid blue curve until a time point between days 5 and 10 when the blue solid curve exceeds the dashed blue curve. This explains the steep decline in graft population initially in the absence of T cells followed by a slower decay than when all T cells are present.

#### Graft Acceptance in the Absence of All T Cells

**Figure 4:**
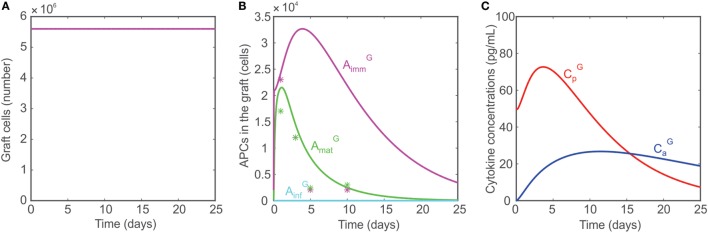
**(A)** Model predicted number of graft cells when no T cells are present. **(B)** Model predicted number of mature APCs (green), immature APCs (magenta), and inflammatory APCs (cyan) in the graft when no T cells are present. The model predictions are compared with data reported in Ref. (40, 41) (asterisks). **(C)** Concentrations of pro-inflammatory cytokines (red) and anti-inflammatory cytokines (blue) when no T cells are present.

As discussed in Ref. (10, 75), animals with no T cells (i.e., no CD4^+^ T cells, no CD8^+^ T cells, and no Tregs) are incapable of rejecting transplants. To simulate conditions of no T cells in the model, the naïve T cell populations are set to 0: T_EN_ = T_RN_ = T_HN_ = 0. As a result, no T cells are generated in the lymph node or graft. Although ${\text{A}}_{\text{mat}}^{\text{G}}$ are activated, the absence of T cells or ${\text{A}}_{\text{inf}}^{\text{G}}$ prevents any damage to the graft (Figure 4A), which survives indefinitely. The APC dynamics in the graft under these conditions are compared with data reported by Oberbarnscheidt et al. (40) in Figure 4B. It shows a fairly accurate description of the trend of ${\text{A}}_{\text{mat}}^{\text{G}}$ with, however, an overestimation of the accumulation of ${\text{A}}_{\text{imm}}^{\text{G}}$, a result that we attribute to the model assumptions employed (see Model Limitations). The levels of the pro- and anti-inflammatory cytokines are shown in Figure 4C.

#### Graft Acceptance in the Absence of T_H_

Several studies (7, 52, 64) have demonstrated that the presence of CD4^+^ T cells is a necessary and sufficient condition for rejection. In accordance with this, the model reproduces the finding that in the absence of CD4^+^ T cells in the lymph node (T_HN_ = 0), the graft is accepted since no damage-inducing cells are activated without the contribution of CD4^+^ T cells.

### Model Simulations

The model is used to assess the effect of altering the number of naïve Tregs (adoptive transfer), altering the translocation rate of T cells from the lymph node to the graft, and performing a transient peri-transplant depletion of T cells. Insight from simulation-generated hypotheses may have eventual implications for designing improved therapeutic strategies that promote tolerance of transplants.

#### Adoptive Transfer of Regulatory T Cells

**Figure 5:**
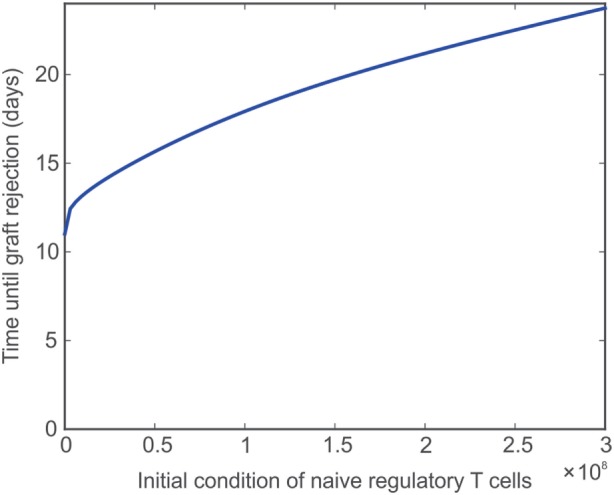
**Impact of naïve Treg adoptive transfer on graft survival**. Model predicted values of time until transplant rejection as the initial (and constant) level of naïve regulatory T cells is varied between 9,500 and 3e8 cells.

Adoptive transfer is a technique by which T cells are obtained from an animal, stimulated in a polyclonal or antigen-specific fashion, and grown in culture. The cells are then transferred back into the original animal or into a separate animal with the overall goal of expanding the frequency of those T cells. Ultimately, this procedure can be exploited to increase or decrease the reactivity of the immune system. Adoptive transfer has been employed using Treg, aiming to counter graft destruction, and is currently under active investigation for its clinical translation (67, 76). The size, frequency, and type of these transfers can vary greatly depending on the system and overall treatment goal. Here, a single injection of naïve Tregs into the lymph node immediately prior to transplantation is simulated by varying T_RN_ from 9,500 cells to 3e8 cells. Figure 5 shows that the graft survival time increases non-linearly with the injection dose. However, fairly rapid transplant rejection is still observed, as expected (67). The model reproduces previous observations that indicate the simple increase of T_RN_ would have a very limited impact on transplant survival unless combined with ideal complementary strategies, such as immunosuppression (in a form that does not affect Treg activity, but only effector T cells) and pre-activation of the injected Tregs (to effectively reduce the levels of the other T cells so that a large ratio of T_R_ to T cells is maintained).

#### Translocation Rates

**Figure 6:**
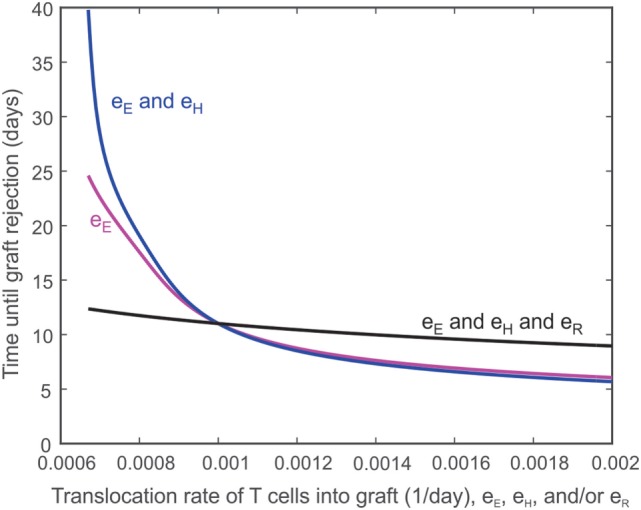
**Impact of T cell translocation rates on graft survival**. Model predicted values of transplant survival times as the translocation rate of CD8^+^ T cells (i.e., e_E_ is varied while the other translocation rates are held constant e_H_ = e_R_ = 0.001 day^−1^, magenta curve) is varied alone or in combination with the translocation rate of CD4^+^ cells (i.e., e_E_ and e_H_ are varied and assumed equal to each other while e_R_ = 0.001 day^−1^, blue curve) or with the translocation of regulatory T cells (i.e., e_E_ and e_H_ and e_R_ are all varied and assumed equal to each other, black curve).

The ease with which T cells can travel between the lymph node and the graft is expected to influence the destruction of the graft. For example, decreasing the rate (e_E_) at which T_E_ cells translocate from the lymph node to the graft should extend the survival of the graft, though not indefinitely. Figure 6 depicts the effect of e_E_ alone on graft survival time (i.e., e_E_ is varied while the other translocation rates are held constant e_H_ = e_R_ = 0.001 day^−1^, magenta curve) or in combination with the translocation rate of CD4^+^ cells (i.e., e_E_ and e_H_ are varied and assumed equal to each other while e_R_ = 0.001 day^−1^, blue curve) or with the translocation rate of Tregs (i.e., e_E_ and e_H_ and e_R_ are all varied and assumed equal to each other, black curve). Under normal model conditions, e_E_ = e_H_ = e_R_ = 0.001 day^−1^. If e_E_ is increased, the graft survival time is decreased from baseline. If both e_E_ and e_H_ are increased, the graft survival time is even more decreased. However, if e_E_, e_H_, and e_R_ are increased, the survival time is longer because more Tregs are present to inhibit the effects of the CD8^+^ and CD4^+^ T cells. The logic is reversed to the left of e_E_ = 0.001 day^−1^.

#### Delayed Injection of T Cells

**Figure 7:**
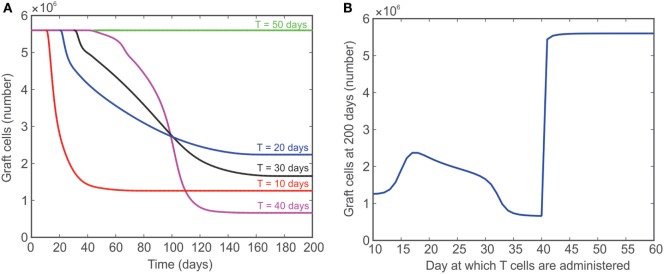
**(A)** Model predicted number of graft cells when no T cells are initially present and then injected after 10 (red), 20 (blue), 30 (black), 40 (magenta), and 50 (green) days. **(B)** The number of graft cells predicted by the model at day 200 when T cells are administered at different time points between 10 and 60 days.

In Figure 7, the model is used to simulate the effect of introducing T cells into a system that originally has no T cells for a fixed number of days [simulations for 10 (red), 20 (blue), 30 (black), 40 (magenta), and 50 (green) days are shown]. These simulations were used to assess the ability of the model to reproduce the outcome of published experiments in which T cells were introduced into a lymphopenic animal 50 days after heart transplantation. The rationale for this test was that the healing process would make the graft incapable of initiating the rejection response. The reported results, however, refuted that hypothesis and showed a complete rejection initiated even when T cells were introduced 50 days after transplant (2). The model presented in the current study fails to predict this outcome, but provides valuable insight into the behavior of the system modeled. For example, the red curve in Figure 7A shows that the model predicts graft acceptance when no T cells are present and graft destruction once T cells are introduced starting at day 10. As indicated by the additional curves in Figure 7A, the steady state population of graft cells (e.g., the population of graft cells after 200 days) does not change monotonically with the number of days till lymphocyte injection. That is, the steady-state number of graft cells is higher if T cells are injected at 20 days instead of 10 days, but lower if T cells are injected at 40 days instead of 10 days. This unexpected behavior is summarized in Figure 7B, which shows the population of graft cells at 200 days as a function of the day at which T cells are injected. This graph clearly shows the non-monotonic relationship between these values.

## Discussion

In this study, a mathematical model of transplant rejection that encompasses both innate and adaptive elements of the immune response is presented. The model is based on combining experimentally observed ratios of different types of T cells in the lymph node and graft as well as the time at which their numbers are maximum together with defined characteristics of the immune response that have been reported in the literature (2–10, 26, 46, 47, 50, 51, 57, 60, 64, 66, 67, 75, 77). Our efforts in the development of this transplant rejection model were driven by its ultimate application as a tool to provide a better understanding of the complex dynamics that underlie the rejection response and to provide a novel and powerful perspective to predict new methods for preventing graft rejection. Three hypothetical immune interventions are explored in this study: modulation of the frequency of naïve Tregs, alteration of the migration of T cells to the graft, and transient depletion of the T cell pool. First, we considered a simple experiment of adoptive transfer of naïve Tregs simulating conditions where the starting number of resting Tregs in the lymph node was altered. Our model indicates that a higher number of Tregs causes an increase in the time to allograft rejection (Figure 5). As expected, however, the impact on graft protection is modest and requires what would be a non-physiological augmentation of Treg numbers to achieve a therapeutic effect. As indicated below, this model is well suited to investigate which combination of strategies could maximize the impact of Treg adoptive transfer (67). For example, although indirectly, the model simulations already suggest a powerful effect of activated Treg migrating to the graft (see comments on third simulation below). While this paper only considers a simple example, the ultimate goal of adoptive transfer is to maintain a high level of Tregs so that they accumulate in both the lymph node and the graft (46, 47, 57, 67). Achieving the greatest possible ratio of Treg to other T cells would yield the maximum inhibitory effect on the activation of ${\text{T}}_{\text{E}}^{\text{G}}$, ${\text{T}}_{\text{H}}^{\text{G}}$, ${\text{C}}_{\text{p}}^{\text{G}}$, ${\text{A}}_{\text{mat}}^{\text{G}}$, and ${\text{A}}_{\text{inf}}^{\text{G}}$, and, as a result, provide a significant protection to the graft.

Second, as shown in Figure 6, reducing the translocation rate of ${\text{T}}_{\text{E}}^{\text{LN}}$ has a non-linear effect on graft destruction. For example, a 50% decrease in e_E_ yields an 82% increase in graft survival time, while a 50% increase in e_E_ decreases the graft survival time by 34%. Decreasing both the translocation rate of ${\text{T}}_{\text{E}}^{\text{LN}}$ and the translocation rate of ${\text{T}}_{\text{H}}^{\text{LN}}$ causes an even more pronounced increase in graft survival time. This protective effect is not only due to a more limited damage inflicted directly by a reduced number of translocating T cells but is also due to the powerful suppressive effect of Tregs that localize to the graft. In fact, the concomitant reduction of e_R_ with e_E_ and e_H_ shows a much more limited prolongation of graft survival. This behavior helps to explain why the inhibition of ${\text{T}}_{\text{E}}^{\text{LN}}$ translocation to the graft has a more beneficial effect than their complete absence (Figure 3). This is probably due to the contribution of ${\text{T}}_{\text{E}}^{\text{LN}}$ to the expansion of Tregs in the lymph node that would then more efficiently control the remaining immune response, a situation that would not occur in the absence of ${\text{T}}_{\text{E}}^{\text{LN}}$. Thus, the manipulation of activated T cell migration could have a more profound therapeutic effect than the prevention of their activation or their deletion, as long as the migration of activated Tregs is not concomitantly affected. Such complex dynamics could contribute to understanding the disparate therapeutic effects observed when targeting specific chemokine receptors (78, 79). Alternatively, this result highlights the importance of using activated Tregs rather than resting ones for adoptive transfer strategies.

Third, the theoretically predicted non-linear and non-monotonic relationship between graft survival and the delayed appearance of alloreactive T cells suggests that new experiments to confirm such a relationship are needed to determine if the results suggest a new method for promoting graft survival. The model prediction is in discordance with the experimental observation that the re-introduction of T cells 50 days post-transplant causes a prompt rejection response (2). This underscores the need to adapt the theoretical model to incorporate other important mechanisms that would contribute to such an outcome. At the same time, this discrepancy indicates that the basic principles implemented in our model are not sufficient to explain the intricate behavior of the immune system and suggest that additional scenarios need to be investigated experimentally. We can speculate two plausible scenarios: (a) the accumulation of pro-inflammatory mediators follows a longer kinetic that supports delayed activation (though not observed experimentally), or (b) the phenomenon of lymphopenia-induced proliferation of T cells (observed when T cells are transferred into a lymphopenic mouse) causes the non-specific activation of T cells that can travel directly to the graft and initiate the rejection response (80). The experimental validation of these possible hypotheses would strengthen the understanding of the non-linear and non-monotonic behavior predicted in this scenario by our model. For example, the model prediction of graft rejection when T cells are administered at 40 days versus the model prediction of near graft acceptance when T cells are administered at 42 days warrants additional investigation. This improved understanding would be essential in determining the extent to which the transient elimination of T cells would be more effective and, possibly, what combinatorial intervention strategy would maximize this effect.

### Model Limitations

Some of the choices and assumptions made in this study limit the capabilities of the model. First, the model focuses on the interactions of T cells, APCs, and inflammatory cytokines, but does not include small-scale details, such as cell signaling or the secretion of various factors. Additionally, cytokines are grouped into two categories (pro- and anti-inflammatory signals) and are tracked only in the graft, not in the lymph node. Considering the vast number of individual cytokine molecules involved in a full immune response as well as their independent dynamics, the relative strength of their effects on the overall immune system (as well as independent effects on individual cell types), and their unique production and decay rates, a model that accounts for each cytokine molecule individually will rapidly become complicated and cumbersome. But, studies show that the overall balance of these signals and their specific varieties can significantly impact graft outcome (4, 6, 33, 47). Effects of pro- and anti-inflammatory cytokines are assumed to be included in parameters such as r_E_, r_R_, and r_H_. The tradeoff of specificity for simplicity allows the model to reproduce general behavior.

Some of the diversity and antigen-specificity of the various cell populations are generally neglected in the model. B cells and memory T cells are excluded, allowing the model to be compared with experimental preparations only involving a naïve T cell repertoire (6, 7, 9, 50, 64, 77, 81). One aspect of initiation of acute organ rejection includes cross-reactivity of non-naïve T cells cross-reacting against specific foreign MHC molecules (HLA in humans) presented by graft cells. The contributions of non-naïve T cells can vary widely, depending on the immunological history of the individual (including the formation of heterologous immunity toward the transplant), and serve as a point of customization that can be adjusted in subsequent iterations of this model. The model also does not accurately represent the accumulation of immature APC in the graft when no T cells are present in the system. This limitation likely derives from the simplification of incorporating multiple APC types into one variable and having ${\text{A}}_{\text{inf}}^{\text{G}}$ and ${\text{A}}_{\text{mat}}^{\text{G}}$ originating from the same starting population. Moreover, the effects of mechanisms of tolerization, namely the induction of T cell anergy by immature APC or the conversion of T cells into Tregs, are not presently included but could be incorporated in the future. These processes contribute significantly to the underlying anti-inflammatory processes, as they allow ${\text{A}}_{\text{imm}}^{\text{G}}$ and ${\text{T}}_{\text{R}}^{\text{G}}$ to inhibit activated cells in ways currently not being modeled. Modeling these factors may require probabilistic considerations of co-stimulatory encounters of antigen with or without pro-inflammatory signals. Additionally, graft rejection experiments typically conclude upon rejection and no further measurements of the graft mass are taken. Thus, any model predictions post-rejection unfortunately cannot be compared to available experimental observations.

The model also assumes that the entire graft is attacked at day 0. In reality, due to the three-dimensional heterogeneity of the system, sites undergoing an inflammatory response are damaged more. There are also early and late inflammatory populations that could be included in the model using a time delay. This would require converting the system into delay differential equations, as in (25).

### Model Extensions

Excitingly, despite the presented limitations, multiple avenues of experimentation to understand the rejection response and to assess the efficacy of therapeutic interventions are suggested by the results obtained with this model. For example, the current model predicts that altering the T_RN_ population has a significant impact on graft survival, as shown in Figures 5 and 6. The size, timing, and repetition of Treg transfers can vary widely; many experiments have started to identify appropriate combinations for maximizing graft life (10, 46, 47, 51, 57, 67, 77). This model can be used to simulate a multitude of adoptive transfer regimens that may or may not have been explored experimentally. In addition, pharmaceutical immunosuppression can be simulated by targeting terms in the equations that represent chemical pathways. In particular, there is much interest in directly manipulating pro- and anti-inflammatory signals as novel immunosuppressive strategies; model simulation could help to identify optimal regimens. The model can also be used to assess the compatibility between the current strategy of immunosuppression and experimental immune interventions and guide the identification of optimal conversion strategies. Moreover, future iterations of the model could encompass the heterogeneity of reactivity of each individual repertoire of alloreactive T cells (combined with the extent of mismatch in HLA molecules between donor and recipient) to achieve a more “personalized” level of intervention – an ideal goal of current medical research. Overall, the model can be used to hypothesize that pathways are viable targets for pharmaceutical intervention based on parameter sensitivity analysis and model dynamics. Combined with a continuous cycle of suggested experimentation and model optimization, this approach has potential for valuable contributions in the quest of transplant tolerance induction.

## Author Contributions

JA, AM, AA, BO, GB, and GR contributed to the theoretical elaboration of the computational model described herein; JA and AM wrote the model script; JA, AM, and AA performed model simulations; BO, GB, and GR executed all wet-lab experiments; JA, AM, AA, BO, GB, and GR wrote the manuscript while providing critical feedback.

## Conflict of Interest Statement

The authors declare that the research was conducted in the absence of any commercial or financial relationships that could be construed as a potential conflict of interest.

## Abbreviations

APCs, antigen-presenting cells; POD, post-operative day; Treg, regulatory T cells.
